# Bis(4,6-diamino­pyrimidin-2-yl) disulfide dimethyl sufoxide disolvate

**DOI:** 10.1107/S160053680802480X

**Published:** 2008-08-09

**Authors:** Ephthymia Papastavrou, Saleh Shakhatreh, Pericles D. Akrivos, Maria Gdaniec

**Affiliations:** a Department of Chemistry, Aristotle University of Thessaloniki, 54006 Thessaloniki, Greece; b Al Huson University College, Al Balqa’ Applied University, Al-Huson, Jordan; c Faculty of Chemistry, Adam Mickiewicz University, 60-780 Poznań, Poland

## Abstract

The title compound, C_8_H_10_N_8_S_2_·2C_2_H_6_SO, was obtained unintentionally during an attempt to prepare a thiol­ate derivative of trimethyl­tin. The complete disulfide mol­ecule is generated by twofold rotation symmetry and the C—S—S—C torsion angle around the S—S bond is −85.70 (10)°. The mol­ecules are connected *via* N—H⋯N hydrogen bonds into strongly corrugated layers parallel to (001), generating an *R*_2_^2^(8) motif. The solvent mol­ecule, which exhibits minor disorder of its S atom [site occupancies = 0.9591 (18) and 0.0409 (18)], is linked to this layer *via* a pair of N—H⋯O inter­actions.

## Related literature

For information on the preferred conformations of organic disulfides, see: Sączewski *et al.* (2006 ▶).
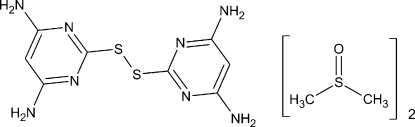


## Experimental

### 

#### Crystal data

C_8_H_10_N_8_S_2_·2C_2_H_6_OS
*M* *_r_* = 438.62
Orthorhombic, 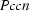

*a* = 11.2612 (4) Å
*b* = 11.9948 (5) Å
*c* = 15.0754 (6) Å
*V* = 2036.32 (14) Å^3^
*Z* = 4
Mo *K*α radiation
μ = 0.49 mm^−1^
*T* = 130 (2) K
0.40 × 0.10 × 0.10 mm

#### Data collection

Kuma KM-4-CCD κ geometry diffractometer
Absorption correction: none
17524 measured reflections
2240 independent reflections
1944 reflections with *I* > 2σ(*I*)
*R*_int_ = 0.035

#### Refinement

*R*[*F* ^2^ > 2σ(*F* ^2^)] = 0.029
*wR*(*F* ^2^) = 0.078
*S* = 1.04
2240 reflections
146 parameters
H atoms treated by a mixture of independent and constrained refinement
Δρ_max_ = 0.23 e Å^−3^
Δρ_min_ = −0.31 e Å^−3^

### 

Data collection: *CrysAlis CCD* (Oxford Diffraction, 2003 ▶); cell refinement: *CrysAlis CCD*; data reduction: *CrysAlis RED* (Oxford Diffraction, 2003 ▶); program(s) used to solve structure: *SHELXS97* (Sheldrick, 2008 ▶); program(s) used to refine structure: *SHELXL97* (Sheldrick, 2008 ▶); molecular graphics: *Stereochemical Workstation Operation Manual* (Siemens, 1989 ▶) and *Mercury* (Macrae *et al.*, 2006 ▶); software used to prepare material for publication: *SHELXL97*.

## Supplementary Material

Crystal structure: contains datablocks global, I. DOI: 10.1107/S160053680802480X/hb2773sup1.cif

Structure factors: contains datablocks I. DOI: 10.1107/S160053680802480X/hb2773Isup2.hkl

Additional supplementary materials:  crystallographic information; 3D view; checkCIF report

## Figures and Tables

**Table 1:** Hydrogen-bond geometry (Å, °)

*D*—H⋯*A*	*D*—H	H⋯*A*	*D*⋯*A*	*D*—H⋯*A*
N7—H7*A*⋯O1^i^	0.78 (2)	2.20 (2)	2.959 (2)	165 (2)
N7—H7*B*⋯N1^ii^	0.846 (19)	2.33 (2)	3.169 (2)	175.1 (16)
N8—H8*A*⋯O1^iii^	0.84 (2)	2.09 (2)	2.9109 (19)	164.4 (18)
N8—H8*B*⋯N3^iv^	0.81 (2)	2.33 (2)	3.088 (2)	156.8 (19)

Symmetry codes: (i) 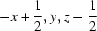; (ii) 
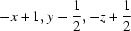
; (iii) 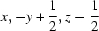; (iv) 
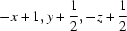
.

**Fig. 1.**
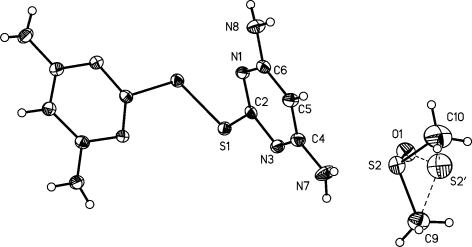
: The molecular structure of (I) with displacement ellipsoids shown at the 50% probability level. Bonds of the DMSO molecule in the minor orientation are shown as dashed lines. The unlabelled atoms of the disulfide molecule are generated by the symmetry operation (3/2-x, 1/2-y, z).

**Fig. 2.**
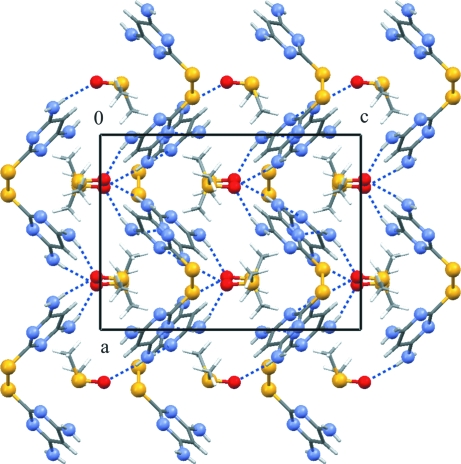
: The crystal structure of (I) viewed down the b axis. Hydrogen bonds are shown with dashed lines.

**Fig. 3.**
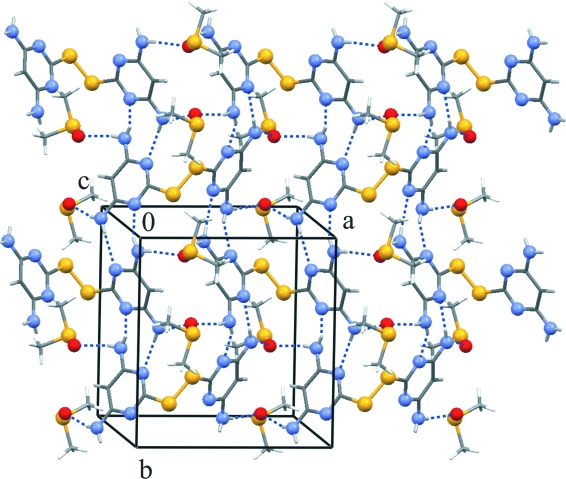
: Hydrogen-bonded two-dimesional assembly of molecules in (I), parallel to the (001) plane. Hydrogen bonds are shown with dashed lines.

## supplementary crystallographic information

### Comment

The title compound, (I), is shown in Fig. 1. In the crystal it adopts a chiral conformation of C_2_ symmetry with the torsion angle around the S–S bond of -85.70 (10)°. The S–S bond length of 2.0249 (7) Å is typical of disulfides in a screw conformation (Sączewski *et al.*, 2006). In turn the torsion angle of -8.23 (13)° around the C–S bond shows that the disulfide S atoms are situated close to the pyrimidine plane.

The component molecules of (I) are connected *via* weak N—H···N interactions generating *R*_2_^2^(8) hydrogen-bond motif into strongly corrugated layer parallel to (001) (Fig. 2, Table 1). The solvent molecules, which exhibit minor disorder of their S atoms, join to this layer *via* a pair of N—H···O interactions and thus all N–H donors are involved in hydrogen bonding. Crystal packing in the title compound is shown in Fig. 3.

### Experimental

1 mmol of 4,6-diaminopyrimidine-2-thiol was dissolved in 10 ml of DMSO at room temperature and was neutralized by the addition of 1 ml of 1 *M* solution of NaOH in methanol. Then, 5 ml of a methanolic solution containing 1 mmol of triorganotin chloride was added and the solution was stirred at room temperature for 1 h. Filtration removed minor solid byproducts and the solution was concentrated by rotary evaporation. After cooling and leaving the solution in the refrigerator for about one week, colourless prisms of (I) appeared.

### Refinement

The S atom of the DMSO molecule is disordered over two positions in a 0.9591 (18):0.0409 (18) ratio. The minor component was refined isotropically.

The H atoms of the N—H groups were located in a difference map and refined isotropically. Positions of the H atoms from the C—H groups were determined geometrically (C—H = 0.96Å) and refined as riding with their isotropic displacement parameters freely refined.

### Crystal data

**Table:**

C_8_H_10_N_8_S_2_·2C_2_H_6_OS	*F*_000_ = 920
*M**_r_* = 438.62	*D*_x_ = 1.431 Mg m^−^^3^
Orthorhombic, *P**c**c**n*	Mo *K*α radiation λ = 0.71073 Å
Hall symbol: -P 2ab 2ac	Cell parameters from 8255 reflections
*a* = 11.2612 (4) Å	θ = 4–27º
*b* = 11.9948 (5) Å	µ = 0.49 mm^−^^1^
*c* = 15.0754 (6) Å	*T* = 130 (2) K
*V* = 2036.32 (14) Å^3^	Prism, colourless
*Z* = 4	0.40 × 0.10 × 0.10 mm

### Data collection

**Table:**

Kuma KM-4-CCD κ geometry diffractometer	1944 reflections with *I* > 2σ(*I*)
Radiation source: fine-focus sealed tube	*R*_int_ = 0.035
Monochromator: graphite	θ_max_ = 27.1º
*T* = 130(2) K	θ_min_ = 4.7º
ω scans	*h* = −14→14
Absorption correction: none	*k* = −15→15
17524 measured reflections	*l* = −19→12
2240 independent reflections	

### Refinement

**Table:**

Refinement on *F*^2^	Secondary atom site location: difference Fourier map
Least-squares matrix: full	Hydrogen site location: difmap and geom
*R*[*F*^2^ > 2σ(*F*^2^)] = 0.029	H atoms treated by a mixture of independent and constrained refinement
*wR*(*F*^2^) = 0.078	*w* = 1/[σ^2^(*F*_o_^2^) + (0.0487*P*)^2^] where *P* = (*F*_o_^2^ + 2*F*_c_^2^)/3
*S* = 1.04	(Δ/σ)_max_ < 0.001
2240 reflections	Δρ_max_ = 0.23 e Å^−^^3^
146 parameters	Δρ_min_ = −0.31 e Å^−^^3^
Primary atom site location: structure-invariant direct methods	Extinction correction: none

### Special details

**Table:**

Geometry. All e.s.d.'s (except the e.s.d. in the dihedral angle between two l.s. planes) are estimated using the full covariance matrix. The cell e.s.d.'s are taken into account individually in the estimation of e.s.d.'s in distances, angles and torsion angles; correlations between e.s.d.'s in cell parameters are only used when they are defined by crystal symmetry. An approximate (isotropic) treatment of cell e.s.d.'s is used for estimating e.s.d.'s involving l.s. planes.
Refinement. DMSO molecule is slightly disordered. In the minor orientation the DMSO C and O atoms superimpose with the C and O atoms of the major orientation. The *S* atom is split into two positions with occupancies 0.96 and 0.04.

### Fractional atomic coordinates and isotropic or equivalent isotropic displacement parameters (Å^2^)

**Table:**

	*x*	*y*	*z*	*U*_iso_*/*U*_eq_	Occ. (<1)
S1	0.69207 (3)	0.18545 (3)	0.35324 (2)	0.01604 (12)	
N1	0.57870 (10)	0.31222 (10)	0.22912 (8)	0.0152 (3)	
C2	0.58248 (12)	0.21471 (12)	0.26959 (9)	0.0144 (3)	
N3	0.51503 (10)	0.12462 (10)	0.25880 (8)	0.0170 (3)	
C4	0.43156 (13)	0.13306 (12)	0.19373 (10)	0.0189 (3)	
C5	0.42109 (13)	0.22991 (12)	0.14298 (10)	0.0198 (3)	
H5A	0.3656	0.2345	0.0948	0.028 (5)*	
C6	0.49402 (13)	0.31911 (12)	0.16390 (10)	0.0166 (3)	
N7	0.36069 (14)	0.04423 (12)	0.18104 (11)	0.0279 (4)	
H7A	0.3204 (18)	0.0445 (16)	0.1393 (13)	0.026 (5)*	
H7B	0.3746 (16)	−0.0163 (16)	0.2079 (11)	0.024 (5)*	
N8	0.48636 (13)	0.41723 (12)	0.11984 (10)	0.0221 (3)	
H8A	0.4314 (19)	0.4234 (16)	0.0824 (13)	0.036 (6)*	
H8B	0.5057 (18)	0.4735 (17)	0.1454 (14)	0.035 (6)*	
S2	0.26010 (4)	0.05431 (4)	0.41883 (3)	0.02342 (15)	0.9591 (18)
O1	0.26997 (10)	0.08760 (10)	0.51429 (7)	0.0276 (3)	
C9	0.21608 (17)	−0.08841 (15)	0.41993 (13)	0.0341 (4)	
H9A	0.2808	−0.1338	0.4402	0.057 (7)*	
H9B	0.1495	−0.0974	0.4591	0.047 (6)*	
H9C	0.1939	−0.1109	0.3611	0.071 (8)*	
C10	0.1254 (2)	0.1121 (2)	0.37893 (17)	0.0564 (7)	
H10A	0.1327	0.1916	0.3738	0.071 (8)*	
H10B	0.1072	0.0809	0.3219	0.104 (11)*	
H10C	0.0628	0.0944	0.4199	0.056 (7)*	
S2'	0.1601 (14)	0.0428 (13)	0.4582 (10)	0.056 (5)*	0.0409 (18)

### Atomic displacement parameters (Å^2^)

**Table:**

	*U*^11^	*U*^22^	*U*^33^	*U*^12^	*U*^13^	*U*^23^
S1	0.01515 (19)	0.01586 (19)	0.0171 (2)	0.00024 (13)	−0.00058 (14)	0.00286 (14)
N1	0.0156 (6)	0.0131 (6)	0.0167 (7)	0.0008 (5)	−0.0011 (5)	−0.0004 (5)
C2	0.0139 (6)	0.0154 (7)	0.0138 (7)	0.0025 (5)	0.0023 (5)	−0.0012 (6)
N3	0.0172 (6)	0.0151 (6)	0.0185 (7)	−0.0009 (5)	−0.0007 (5)	−0.0001 (5)
C4	0.0186 (7)	0.0161 (7)	0.0221 (8)	0.0002 (6)	−0.0010 (6)	−0.0024 (6)
C5	0.0201 (7)	0.0168 (8)	0.0226 (8)	0.0006 (6)	−0.0058 (6)	−0.0014 (6)
C6	0.0179 (7)	0.0158 (7)	0.0162 (7)	0.0024 (5)	0.0010 (6)	−0.0013 (6)
N7	0.0306 (8)	0.0179 (7)	0.0353 (9)	−0.0077 (6)	−0.0162 (7)	0.0053 (7)
N8	0.0252 (7)	0.0164 (7)	0.0247 (8)	−0.0013 (6)	−0.0099 (6)	0.0019 (6)
S2	0.0229 (2)	0.0294 (3)	0.0179 (2)	0.00107 (17)	−0.00281 (17)	0.00052 (17)
O1	0.0298 (6)	0.0316 (7)	0.0215 (6)	0.0015 (5)	−0.0053 (5)	−0.0091 (5)
C9	0.0388 (10)	0.0317 (10)	0.0318 (11)	−0.0018 (8)	−0.0077 (8)	−0.0085 (8)
C10	0.0490 (14)	0.0504 (14)	0.0697 (17)	0.0079 (11)	−0.0347 (13)	0.0077 (13)

### Geometric parameters (Å, °)

**Table:**

S1—C2	1.7990 (15)	N7—H7B	0.846 (19)
S1—S1^i^	2.0249 (7)	N8—H8A	0.84 (2)
N1—C2	1.3198 (18)	N8—H8B	0.81 (2)
N1—C6	1.3722 (19)	S2—O1	1.4976 (11)
C2—N3	1.3309 (19)	S2—C10	1.773 (2)
N3—C4	1.3623 (19)	S2—C9	1.7822 (19)
C4—N7	1.345 (2)	C9—H9A	0.9600
C4—C5	1.396 (2)	C9—H9B	0.9601
C5—C6	1.385 (2)	C9—H9C	0.9601
C5—H5A	0.9601	C10—H10A	0.9601
C6—N8	1.354 (2)	C10—H10B	0.9601
N7—H7A	0.78 (2)	C10—H10C	0.9600
			
C2—S1—S1^i^	107.03 (5)	C6—N8—H8A	116.9 (13)
C2—N1—C6	114.02 (12)	C6—N8—H8B	118.4 (14)
N1—C2—N3	130.14 (13)	H8A—N8—H8B	116 (2)
N1—C2—S1	121.27 (11)	O1—S2—C10	106.58 (10)
N3—C2—S1	108.59 (10)	O1—S2—C9	105.54 (8)
C2—N3—C4	114.92 (12)	C10—S2—C9	98.08 (11)
N7—C4—N3	116.94 (14)	S2—C9—H9A	109.7
N7—C4—C5	122.08 (15)	S2—C9—H9B	109.3
N3—C4—C5	120.98 (13)	H9A—C9—H9B	109.5
C6—C5—C4	117.90 (14)	S2—C9—H9C	109.5
C6—C5—H5A	120.9	H9A—C9—H9C	109.5
C4—C5—H5A	121.2	H9B—C9—H9C	109.5
N8—C6—N1	116.62 (13)	S2—C10—H10A	110.0
N8—C6—C5	121.46 (14)	S2—C10—H10B	109.5
N1—C6—C5	121.91 (13)	H10A—C10—H10B	109.5
C4—N7—H7A	117.4 (15)	S2—C10—H10C	108.9
C4—N7—H7B	120.2 (12)	H10A—C10—H10C	109.5
H7A—N7—H7B	119.9 (19)	H10B—C10—H10C	109.5
			
C6—N1—C2—N3	−2.4 (2)	C2—N3—C4—C5	0.6 (2)
C6—N1—C2—S1	176.75 (10)	N7—C4—C5—C6	176.79 (15)
S1^i^—S1—C2—N1	−8.23 (13)	N3—C4—C5—C6	−3.3 (2)
S1^i^—S1—C2—N3	171.09 (8)	C2—N1—C6—N8	−179.77 (13)
N1—C2—N3—C4	2.5 (2)	C2—N1—C6—C5	−0.8 (2)
S1—C2—N3—C4	−176.76 (10)	C4—C5—C6—N8	−177.67 (14)
C2—N3—C4—N7	−179.45 (14)	C4—C5—C6—N1	3.4 (2)

Symmetry codes: (i) −*x*+3/2, −*y*+1/2, *z*.

### Hydrogen-bond geometry (Å, °)

**Table:**

*D*—H···*A*	*D*—H	H···*A*	*D*···*A*	*D*—H···*A*
N7—H7A···O1^ii^	0.78 (2)	2.20 (2)	2.959 (2)	165 (2)
N7—H7B···N1^iii^	0.846 (19)	2.33 (2)	3.169 (2)	175.1 (16)
N8—H8A···O1^iv^	0.84 (2)	2.09 (2)	2.9109 (19)	164.4 (18)
N8—H8B···N3^v^	0.81 (2)	2.33 (2)	3.088 (2)	156.8 (19)

Symmetry codes: (ii) −*x*+1/2, *y*, *z*−1/2; (iii) −*x*+1, *y*−1/2, −*z*+1/2; (iv) *x*, −*y*+1/2, *z*−1/2; (v) −*x*+1, *y*+1/2, −*z*+1/2.
